# Research progress of two-pore potassium channel in myocardial ischemia-reperfusion injury

**DOI:** 10.3389/fphys.2024.1473501

**Published:** 2024-10-29

**Authors:** Yue Zhang, Jing Li, Jiamei Pan, Shengli Deng

**Affiliations:** Department of Anesthesiology, The Affiliated Hospital of Zunyi Medical University, Zunyi, Guizhou, China

**Keywords:** two-pore potassium channel, K2P channel, KCNK, cardiovascular disease, myocardial ischemia-reperfusion injury

## Abstract

Myocardial ischemia-reperfusion injury (MIRI) is a secondary injury caused by restoring blood flow after acute myocardial infarction, which may lead to serious arrhythmia and heart damage. In recent years, the role of potassium channels in MIRI has attracted much attention, especially the members of the two-pore domain potassium (K2P) channel family. K2P channel has unique structure and function, and the formation of its heterodimer increases its functional diversity. This paper reviews the structural characteristics, types, expression and physiological functions of K2P channel in the heart. In particular, we pay attention to whether members of the subfamily such as TWIK, TREK, TASK, TALK, THIK and TRESK participate in MIRI and their related mechanisms. Future research will help to reveal the molecular mechanism of K2P channel in MIRI and provide new strategies for the treatment of cardiovascular diseases.

## 1 Introduction

Cardiovascular disease is the leading cause of death worldwide (Cardiovascular diseases), of which ischemic heart disease has the highest mortality risk (NCD Countdown, 2030 collaborators, 2020; Rea, 2024). This condition is usually caused by stenosis or obstruction of the coronary artery, leading to ischemia and hypoxia of the myocardial tissue, which may eventually evolve into acute myocardial infarction (Bae et al., 2021). At present, timely reperfusion therapy is the key to save the lives of patients with acute myocardial infarction, including coronary artery bypass grafting, percutaneous coronary intervention, and drug thrombolysis (Ibanez et al., 2018; Völz et al., 2021). However, cardiomyocytes may encounter myocardial ischemia-reperfusion injury (MIRI) when blood flow recovers to an oxygen-rich environment, which may induce severe arrhythmia and even heart damage such as ventricular fibrillation (Wang et al., 2014; Rodrigue et al., 2022; Yellon and Hausenloy, 2007). When MIRI occurs, endothelial cells, immune cells, fibroblasts, and other cells are stimulated and engage in a series of complex pathophysiological reactions (Eltzschig and Eckle, 2011). The mechanisms involved encompass oxidative stress, inflammatory response, calcium overload, and mitochondrial dysfunction (Zodda et al., 2023; Zhuang et al., 2023; Zhang et al., 2023; Tu et al., 2020).

In the research on the mechanism of MIRI, scholars have found that potassium ion channel plays a vital role. The potassium channels were mainly divided into four categories: voltage-gated potassium channel, calcium-dependent potassium channel, inward-rectified potassium channel and two-pore domain potassium (K2P) channel (Zhang et al., 2022; Dupuy et al., 2023). Among them, K2P channel, as a newly discovered family of potassium channels, has been confirmed to have an important target value in the treatment of cardiovascular diseases. In this paper, the current status of research on the K2P channel in MIRI is systematically reviewed, in order to provide a theoretical basis and a new perspective for the development of relevant treatment strategies.

## 2 Structural types, characteristics and physiological functions of K2P channel

K2P channel, as an important member in the potassium channel family, has attracted much attention due to their molecular structure and functional characteristics. It was divided into six subfamilies: TWIK (Tandem of P domains in a weak inward rectifying K^+^ channel), TREK (TWIK-related K^+^ channel), TASK (TWIK-related acid-sensitive K^+^ channel), TALK (TWIK-related alkaline pH-activated K^+^ channel), THIK (tandem pore domain halothane-inhibited K^+^ channel) and TRESK (TWIK-related spinal cord K^+^ channel). These subfamilies are further subdivided into 15 distinct channel subtypes, each encoded by KCNK1 through KCNK18 genes (Enyedi and Czirják, 2010; Sepúlveda et al., 2015) (Table 1).

**TABLE 1:** Evidence for the association of K2P channels with MIRI.

The K2P subfamily		Correlation with MIRI	Citation
TWIK	TWIK-1 (KCNK1, K2P1.1)	Low PH or low potassium conditions: dynamic ion selectivity	Ma et al. (2011, 2012)
		As a Bayesian network for predicting coronary artery calcification	McGeachie et al. (2009)
		The expression is up-regulated in Brugada syndrome	N et al. (2009)
		Rat heart failure model: increased expression in the sinoatrial node	Yanni et al. (2011)
	TWIK-2 (KCNK6, K2P6.1)	It is involved in the activation of macrophage inflammasomes	Di et al. (2018)
		HCF was more expressed in mechanosensitive ion channels	Mitrokhin et al. (2023)
	TWIK-3 (KCNK7, K2P7.1)	Expression was upregulated in AF	Wang et al. (2018)
TREK	TREK-1 (KCNK2, K2P2.1)	Mouse coronary artery ligation model: alleviation of MIRI	Kamatham et al. (2019)
		Rat myocardial infarction model: decreased expression in the infarct area and increased expression in the infarct border area	Ln et al. (2011)
		Neonatal rat model of acute myocardial infarction: increased expression and inhibited cardiomyocyte proliferation	Yang et al. (2014)
		Foxd3/miR-214/Kcnk2 axis, which is involved in the occurrence of SCII, is down-regulated. When its expression is increased, it plays a protective role in SCII	Li et al. (2020b)
		Rat model of ventricular hypertrophy: upregulated expression in the endocardium plays a protective role in the heart	Wang et al. (2013)
		Pressure overload model: specific loss of cardiomyocytes caused cardiac dysfunction; Specific deletions in fibroblasts were protective	Abraham et al. (2018)
	TREK-2 (KCNK10, K2P10.1)	Rat middle cerebral artery occlusion model: downregulation of expression attenuated the protective effect of isoflurane preconditioning	Zhao et al. (2019)
	TRAAK (KCNK4, K2P4.1)	Knockout mice survived healthy after cerebral infarction without obvious arrhythmia	Lloyd et al. (2011), Laigle et al. (2012)
TASK	TASK-1 (KCNK3, K2P3.1)	In knockout mice, QRS complex was widened and QT interval was prolonged. After isoflurane anesthesia, there was no change in the gene knockout mice, and the heart rate was reduced and the PR interval was prolonged in the wild mice	Pandit et al. (2020)
		Rat diabetic model: upregulated expression and decreased heart rate in sinoatrial node	Ferdous et al. (2016)
		Mean heart rate was elevated in TASK1 knockout mice	Donner et al. (2011)
		The specific marker of lung pericytes, TASK-1, was highly expressed	Sh et al. (2022)
		Pressure overload model: gene knockout mice have good new function and reduced cardiac hypertrophy	Duan et al. (2020)
		In patients with sinus rhythm: decreased atrial expression correlates with left ventricular dysfunction	Schmidt et al. (2017)
		Rat model of diabetic cardiomyopathy: after ALDH2 is activated, TASK-1 acts as its downstream target to protect the heart	Zhang et al. (2019)
	TASK-3 (KCNK9, K2P9.1)	Stress overload model: TASK3 knockout mice had cardiac hypertrophy and reduced onset time of cardiac dysfunction	Duan et al. (2020)
	TASK-5 (KCNK15, K2P15.1)	Unknown	NA
TALK	TALK-1 (KCNK16, K2P16.1)	Unknown	NA
	TALK-2 (TASK-4、KCNK17, K2P17.1)	The expression level was higher in non-ischemic heart failure tissues than in ischemic heart failure tissues	Chai et al. (2017)
	TASK-2 (KCNK5, K2P5.1)	It is activated by ROS and is involved in the volume reduction during renal cell apoptosis	L’Hoste et al. (2007)
		Dysregulation of expression in AF	Mondéjar-Parreño (2022)
		In diabetic rat model, heart rate decreased and KCNK5 expression was down-regulated	Howarth et al. (2018)
		It can be involved in the regulation of PITX2 on RMP, and can be used as a target for antiarrhythmic drug treatment	Syeda et al. (2016)
THIK	THIK-1 (KCNK13, K2P13.1)	IL-1β release from microglia was reduced after gene knockout	Gardener et al. (2004)
	THIK-2 (KCNK12, K2P12.1)	Unknown	NA
TRESK	KCNK18	Unknown	NA

Literature evidence for the association of K2P channel subunits with MIRI. HCF, cardiac fibroblasts; SSCI, spinal cord ischemia-reperfusion injury; Foxd3, forkhead box transcription factor D3; ALDH2, aldehyde dehydrogenase 2; PITX2, a paired homeodomain-2, transcription factor; RMP, resting membrane potential.

**FIGURE 1:**
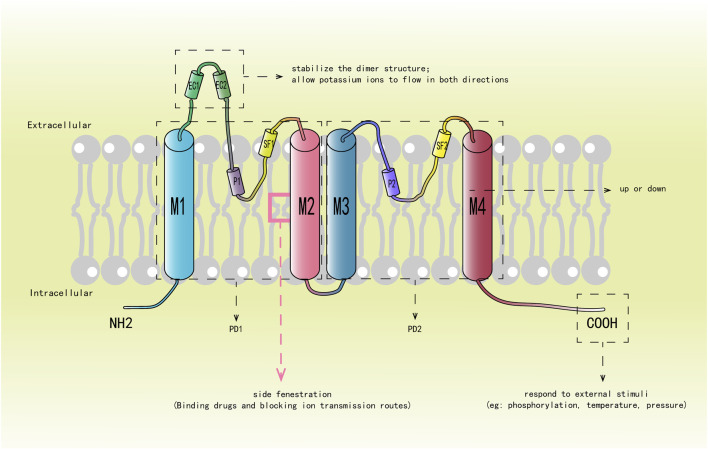
Describes the structure of each subunit and the function of some important components. M1-M4 represent four transmembrane helices, M1 and M3 are outer helices, which are shown in blue, and M2 and M4 are inner helices, which are shown in red. P1 and P2 represent two pore domains; SF1 and SF2 denote selectivity filters; EC1 and EC2 constitute the hat domain, and the red box represents the “side fenestration” generated by M2.

K2P channel is composed of two subunits, each of which includes four transmembrane helices (M1, M2, M3 and M4) arranged in series, two pore helices (P1 and P2) formed by membrane reentrant regions on both sides, two selectivity filters (SF1 and SF2) and an extracellular caps domain (consisting of EC1 and EC2). Each subunit forms two pore-forming domains (PD1 and PD2), which are connected to each other through the M2 transmembrane helix segment. Figure 1 shows the structure of each subunit and the function of some important components: PD1 is composed of M1, M2, SF1 and P1; PD2 is consisted of M3, M4, SF2 and P2. As outer spirals, M1 and M3 are perpendicular to the SF in the membrane; While M2 and M4 act as inner spirals, are located on the bevel below the SF (Mathie et al., 2021; Dong et al., 2015; Brohawn et al., 2012; Lin et al., 2024; Bustos et al., 2020). On the intracellular side, the amino terminal of M1 is shorter. In contrast, M4 has a long carboxyl terminal and contains various regulatory phosphorylation sites and protein interaction motifs. P1 and P2 are suspended in the channel pores like the bassinet. EC1 and EC2 have a unique cap structure formed by conservative α helices, protruding outward to form tunnel like side grids on the membrane, becoming a significant structural feature of K2P channels. Most K2P channel family members have conservative cysteine residues at the top of the cap structure, forming disulfide bonds to stabilize the dimer structure between subunits. The TASK and THIK channels lack cysteine at these positions, and the stability of their dimer structure is mainly depended on the interaction of the hydrophobic residues in the cap domain. The extracellular cap domain participates in regulating the opening and closing of the channel, allowing the bidirectional flow of potassium ions and preventing toxins from entering the channel hole from above or laterally (Brohawn et al., 2012; Rödström et al., 2020; Miller and Long, 2012).

The SF is composed of a GYG (glycine-tyrosine-glycine) sequence that imparts ion selectivity to the channel (Enyedi and Czirják, 2010; Dong et al., 2015). Directly below it is the “side fenestration” of the M2 transmembrane segment formed by bending about 20° at the center of the membrane, which is highly conservative (Brohawn et al., 2012; Miller and Long, 2012; Aryal et al., 2014). The M4 helix can directly affect the opening and closing of the channel through its conformational change, which is the so-called “c-type” gating mechanism (Schewe et al., 2016; Zhuo et al., 2016). The carboxyl terminal of M4 feels the stimulation of phosphorylation, temperature and pressure, and controls the opening and closing of the “side fenestration” by increasing or decreasing changes, thereby causing the conformational change of the SF and affecting the conductivity of the channel (Schewe et al., 2016; Türkaydin et al., 2024; Sn et al., 2011; Feliciangeli et al., 2015; Douguet and Honoré, 2019; Piechotta et al., 2011; Lolicato et al., 2014). In the ascending state, M4 is oriented towards the bilayer of the membrane, and the “side fenestration” in the membrane is closed. The SF is conformationally stable and allows potassium ions to pass through. In the descending state, M4 is away from the intima, and the side fenestration is opened. The conformational change of SF leads to inactivation and prevented the passage of potassium ions (Zhang et al., 2022; Dong et al., 2015; Türkaydin et al., 2024; Lolicato et al., 2014; Stephen et al., 2013; Brohawn et al., 2014). Therefore, it is also a key target for channel inhibitors to exert their effects. Subsequent studies have proposed that the decline of M4 is not a complete closure of the channel, but an intermediate state, which still has a certain conductivity, while the conductivity is better when M4 rises (Zhang et al., 2022; Lolicato et al., 2014; McClenaghan et al., 2016; Proks et al., 2021; Brennecke and de Groot, 2018). Therefore, the combination of K2P channel and drug mainly occurred at the “side fenestration” under the SF, blocking the ion conduction pathway in the cell lumen (Dong et al., 2015; Rödström et al., 2020).

The difference of each subunit PD1 and PD2 determines its formation of homodimer or heterodimer. Two different subunits form a heterodimer, which can change or generate new channel characteristics, thus increasing the functional diversity. At present, the heterodimers are composed of subunits of members of the same subfamily include: TASK-1/TASK-3, TASK-1/TASK-3/TASK-5, TREK-1/TREK-2, TREK-1/TRAAK, TREK-2/TRAAK, TALK-1/TALK-2/TASK-2 and THIK-1/THIK-2 (Berg et al., 2004; Rinné et al., 2015; Czirják and Enyedi, 2002; Blin et al., 2014; Lengyel et al., 2016; Watanabe et al., 2016; Levitz et al., 2016; Khoubza et al., 2022). Heterodimers are consisted of subunits of different subfamilies in K2P channel include: TWIK-1/TASK-1, TWIK-1/TASK-3, TWIK-1/TREK-1, TWIK-1/TREK-2, TASK-1/TALK-2, TRESK/TREK-1/TREK-2 and TWIK-1/TASK-1/TASK-3 (Choi et al., 2018; Hwang et al., 2014; Suzuki et al., 2017; Royal et al., 2019; Lengyel et al., 2020; Plant et al., 2012; Rinné et al., 2024).

Leakage current generated by K2P channel causes outward flow of potassium ions, causing hyperpolarization of the cell membrane and thus maintaining resting membrane potential of the cell (Feliciangeli et al., 2015; Nomura et al., 2015; Huang et al., 2022; Ryoo and Park, 2016). K2P channel is affected by a variety of endogenous and exogenous stimulation factors, including temperature, voltage, membrane stretching, PH, signaling protein, lipids and glycosylation (Schewe et al., 2016; Feliciangeli et al., 2015; N et al., 2021; Mant et al., 2013; Wiedmann et al., 2019). The complexity and regulatory mechanism of these channels are of great significance to the maintenance of cell function and adaptation to different physiological conditions.

### 2.1 TWIK and MIRI

The TWIK subfamily of K2P channel consists of three members, TWIK-1, TWIK-2, and TWIK-3.

TWIK-1 (K2P1.1 or KCNK1) is the first identified channel in this family, and its expression has been found in human brain and heart (Lesage et al., 1996; Arrighi et al., 1998). Particularly, in cardiac tissue of human, TWIK-1 mRNA expression was highest in the atrium (Caroline et al., 2007; Christensen et al., 2016; Gaborit et al., 2007; Balázs et al., 2006). However, early studies using RT-PCR and Northern blot indicated that TWIK-1 was more expressed in the human ventricle (Lesage et al., 1996; Z et al., 1998). In the heterologous expression system, the functional activity of the TWIK-1 under physiological conditions was confirmed to be very low (Rajan et al., 2005), this phenomenon is mainly attributed to its SF conductive conformation has obvious instability (Nematian-Ardestani et al., 2020; Chatelain et al., 2012). This structural property limits the function of the TWIK-1 channel in the regulation of cell membrane potential, but its conservative role in the heart cannot be ignored. Such conservatism of TWIK-1 may be related to its fine regulation in cardiac physiological function, which helps maintain the normal rhythm of the heart and stabilize the atrial structure (Christensen et al., 2016). The activity of TWIK-1 in the cardiac was blocked by quinine, quinidine, and Ba^2+^compounds (Schmidt et al., 2017a). In addition, magnolol is a natural compound having anti-inflammatory and cardiovascular protective effects (Chen et al., 2019; Jin et al., 2021; Karki et al., 2013), can not only improve ischemic stroke (Liu et al., 2018), but also has been found to inhibit the activity of TWIK-1 in HEK293 cells with heterologous expression system (Wang et al., 2023). Metabotropic glutamate receptor three can activate the astrocyte TWIK-1 and upregulate its protein expression, thus promoting the uptake of ammonia ion by astrocytes (Wang et al., 2016).

TWIK-1 is a pH-gated potassium channel that responds to extracellular acidic environments (Rajan et al., 2005; Turney et al., 2022). At high pH, the TWIK-1 channel is open; At low pH, protonation of extracellular histidine will cause conformational changes. These changes will seal the top of the SF, replace the screw cap to block the extracellular ion channel and open the gap for lipid block in the cell cavity to close the channel (Turney et al., 2022). Studies have demonstrated that TWIK-1 exhibits dynamic ion selectivity. When extracellular pH decreases or hypokalemia occurs, the permeability of extracellular sodium ions is enhanced, leading to increased sodium influx and inducing membrane depolarization (Ma et al., 2011; Ma et al., 2012). Clinical studies have shown that patients undergoing coronary intervention are susceptible to hypokalemia, which significantly increases the risk of arrhythmia (Qian et al., 2023; Ma Y. et al., 2022). In the early stage, Michael McGachie et al. ^83^proposed that KCNK1 can be used as a Bayesian network for predicting the presence of coronary artery calcification, which can better predict disease models, but no further research has been conducted. The pathophysiological process of coronary intervention is involved in MIRI, and hypokalemia is prone to occur after coronary artery therapy. TWIK-1 shows dynamic ion selectivity in hypokalemia, but whether TWIK-1 is involved in MIRI by regulating ion flow has not been reported. In addition, clinical studies have found that the expression of TWIK-1 is relatively upregulated in patients with Brugada syndrome, suggesting that TWIK-1 may play a specific role in the arrhythmia of Brugada syndrome (such as idiopathic ventricular fibrillation) (Nathalie et al., 2009). However, there is a lack of relevant studies to clarify the mechanism. Brugada syndrome, a genetic disorder with associated risk of idiopathic ventricular fibrillation and of consequently sudden death, is mainly thought to be related to the decrease of sodium and calcium influx or the increase of potassium outflow from the myocardium, which involves in the depolarization and repolarization of action potential (Brugada et al., 2018). Among them, the primary repolarization disorder caused by shortening of epicardial action potential duration (APD) has been widely studied (Di Diego et al., 1996). TWIK-1 mainly generates an outward potassium current to maintain the resting potential. The expression of TWIK-1 is increased in the heart of patients with Brugada syndrome, which may suggest that TWIK-1 is involved in the occurrence and development of repolarization disorder in Brugada syndrome by promoting potassium efflux, shortening APD, and leading to idiopathic ventricular fibrillation. However, the specific pathophysiological process and related pathway mechanisms are still unexplored. Yanni et al., (2011) found increased TWIK-1 expression in the sinoatrial node in a rat model of heart failure, suggesting its role in heart rate regulation. At present, it is speculated that the possible mechanism is that TWIK-1 increases potassium efflux in cardiac sinoatrial node cells when they are at resting potential, causing cell hyperpolarization, which leads to endogenous slowing of heart rate. It is concluded that TWIK-1 plays a role in the cardiac conduction system and heart rate regulation, and is involved in the occurrence of arrhythmia.

TWIK-2 (K2P6.1 or KCNK6) is widely expressed in various tissues of the human body, with the highest expression level particularly in the pancreas (Chavez et al., 1999), while it is less expressed in the heart and mainly expressed in the right atrium (Liu and Saint, 2004; Wiedmann et al., 2018; Howarth et al., 2018). TWIK-2 is also expressed in rat heart (Yanni et al., 2011; Howarth et al., 2018; Bobak et al., 2017). There is relatively little functional research on TWIK-2, but its selective inhibitors are known to include ML365 and NPBA (Wu et al., 2022; Zhi et al., 2023). TWIK-2 gene knockout prevents activation of NLRP3 inflammasomes in bone marrow-derived macrophages (Di et al., 2018), suggesting that TWIK-2 might play a role in the inflammatory response. The gap junction between human cardiac fibroblasts (HCF) and cardiomyocytes is involved in the regulation of cardiac electrophysiology and plays an important role in regulating cardiac excitability (Mahoney et al., 2016; Ieda et al., 2009; Cai et al., 2023). Recent studies have reported that gap junction protein combined with ischemic preconditioning can reduce the infarct size after MIRI in rats (Aleksander et al., 2022). In exploring the mechanosensitive ion channel (MscL) group of human HCF, the K2P channel family is the second most expressed cation channel family, with the TWIK-2 being the most abundant (Mitrokhin et al., 2023). However, whether TWIK-2 affects gap junction proteins through HCF to participate in the pathophysiological process of MIRI has not been reported.

Limited research has been conducted on TWIK-3 (K2P7.1 or KCNK7). However, TWIK-3 mRNA has been shown to be expressed in the human heart and upregulated in patients with atrial fibrillation (AF) (Wang et al., 2018). This suggests that TWIK-3 may be related to the occurrence and development of heart disease, but its specific mechanism and function need further investigation.

### 2.2 TREK and MIRI

The TREK subfamily consists of three members: TREK-1, TREK-2, and TRAAK (Kim et al., 2023). These channels share a high degree of structural similarity. TREK-2 and TREK-1 share 63% identity and 78% homology in amino acid sequence. In contrast, TRAAK and TREK-1 have a lower identity of 45% and a homology of 69%, indicating significant differences in their sequences (Xue et al., 2020). The application of patch-clamp technique revealed the characteristics of TREK channel as a low-threshold mechanically sensitive channel, and their responses to changes in cell membrane tension were different: TRAAK was the most sensitive, TREK-1 was in the middle, and TREK-2 had the lowest sensitivity. TRAAK and TREK-1 can be activated by all physiologically relevant stresses, and low-frequency, low-intensity focused ultrasound can activate TRAAK and mesenchymal stem cells (MscS) by increasing membrane tension. In contrast, the activation of TREK-2 requires more specific conditions, such as the involvement of mechano-gated cation channel family members, MscS and mechano-sensitive channels such as MscL (Ben et al., 2024).

In the aspects of cell signaling and functional regulation, the specific protein-protein interaction is essential to maintain the stability of the intracellular environment. Microtubule-associated protein 2 and a-kinase ankyrin 150 have been shown to interact with and positively regulate the expression of the TREK-1 and TREK-2 (Sukharev, 2002; Sandoz et al., 2006). This interaction not only plays a key role in the intracellular localization of the channel, but also may affect its functional expression on the cell membrane. In addition to the protein interactions, certain small molecule compounds have also been found to modulate the activity of the TREK channel. For example, both BL1249 and the clinically used inhalation anesthetic halothane have the ability to activate the TREK-1 and TREK-2 channels (Cox et al., 2016). This indicated that these compounds might change their conformational states by directly or indirectly acting on the channel proteins, thus adjusting their opening probability and ion conductivity. However, not all compounds had the same effect on TREK channel subtypes. Ruthenium red, as a known ion channel modulator, is able to specifically inhibit the activity of the TREK-2 and TRAAK (Lewis and Grandl, 2015; Braun et al., 2015). The characteristics of this selective action suggest that different TREK channel subtypes may have different pharmacological properties and regulatory mechanisms.

TREK-1 (KCNK2 or K2P2.1) is widely expressed in a variety of tissues, especially in human, rat, mouse, and porcine heart tissues (Abraham et al., 2018; Darkow et al., 2023; Joy et al., 2004; Xian Tao et al., 2006; Schmidt et al., 2017b). Notably, TREK-1 is abundantly expressed in the heart, but its expression levels vary in various sites among different species. In normal human and heart tissues from patients with heart disease, TREK-1 was found to be more expressed in the ventricle than in the atrium (Darkow et al., 2023; Schmidt et al., 2015). In the rat heart, TREK-1 content was highest in the left ventricle. The expression of TREK-1 in the endocardium was 17 times higher than that in the epicardium, and the action potential shortening was more significant in the endocardium (Joy et al., 2004; Wang et al., 2013). In the mouse heart, TREK-1 is mainly expressed in the left ventricle, and less expressed in the atrium (Liu and Saint, 2004; Wiedmann et al., 2018; Schmidt et al., 2017b). However, it has also been shown that the expression of TREK-1 mRNA in mouse heart is mainly in the atrium (Unudurthi et al., 2016). TREK-1 channels were abundantly expressed in the porcine heart, which showed 99% similarity with the TREK-1 protein in the human heart. However, more TREK-1 channels were detected in the atrium than in the ventricle (Schmidt et al., 2014a). In addition, TREK-1 mRNA expression was significantly higher in the apex than in the bottom ventricle (Kelly et al., 2006). The expression of TREK-1 varies in different parts of the heart among different species, suggesting that TREK-1 may play diverse physiological and pathological roles in heart diseases.

The activity of TREK-1 channel can be regulated by a variety of factors. Volatile anesthetics, intracellular acidification, arachidonic acid (AA), and mechanical stretching all activate TREK-1 (Schmidt et al., 2017b; Wang et al., 2013; Kanda et al., 2021). Adrenocorticotropic hormone, angiotensin II, L-3-n-butylphthalide and hairy throat can inhibit TREK-1 (Wang et al., 2013; Enyeart and Enyeart, 2021; Terrenoire et al., 2001). Recent studies have revealed the regulatory effect of the antidepressant peptide spadin, as a specific blocker, on the function of the TREK-1 channel. Studies have shown that the activity of the TREK-1 channel in mice may undergo significant changes after pre-exposuring to spadin, which is likely achieved through a deformation mechanism, thus disrupting the subsequent activation of TREK-1 current by AA Ma and Lewis (2020). Lv et al. (2024) found a significant reduction in the differential metabolite AA using a non-targeted UPLC-qTOF-MS metabolomics approach through a porcine diabetic myocardial infarction model. Subsequently, by using an isolated diabetic myocardial ischemia model, the authors found a decrease in intracellular AA levels and an increase in cardiomyocyte apoptosis. The production of intracellular and mitochondrial ROS (mt ROS) was reduced and mitochondrial function was improved by the addition of exogenous AA. Activation of PINK1-Parkin-dependent mitophagy may be responsible for this phenomenon. Finally, coronary artery ligation was performed in diabetic Sprague Dawley rats to establish an *in vivo* diabetic myocardial ischemia model. After AA treatment, the myocardial infarct size, apoptosis index and inflammatory cytokines were reduced, and the myocardial fiber fracture and light and dark band fusion were also significantly improved. At the same time, the authors collected and compared the venous blood of healthy patients, diabetic patients and non-diabetic patients after presenting to the emergency department with symptoms of myocardial infarction. The results showed that the blood AA level was lower and the cardiac ejection fraction was lower in diabetic patients with myocardial infarction than in the other two groups, which again proved that AA metabolism was the key to myocardial ischemic injury in diabetes. In addition, AA scoring systems based on AA metabolite production have been developed to predict the risk of death in patients with acute decompensated heart failure (Ma K. et al., 2022; Miyauchi et al., 2024; S W. et al., 2016). The application of this scoring system provides clinicians with a powerful tool to assess and monitor the progression of heart failure patients and the efficacy of treatment. However, there is currently no research report on whether AA participates in MIRI by affecting TREK-1.

TREK-1 is a mechanosensitive potassium channel (Ben et al., 2024). Studies have confirmed that mechanoelectric feedback (MEF) of mechanosensitive ion channels can affect the electrophysiological activity of the heart after sensing the membrane stretch (De et al., 1990). Jose A Barrabés et al. (2002) established a porcine model of ligation of the left anterior descending coronary artery and showed an increase in end-diastolic length of the ischemic zone measured by ultrasound crystals. They concluded that this increase in length was independently and closely related to ventricular fibrillation after coronary artery occlusion. In the rat myocardial infarction model, Kiseleva et al. (2000) found that in the infarct junction region, the degree of membrane stretch is lower and the myocardial cell membrane potential is more sensitive to mechanical stimulation, which leads to a higher risk of ventricular arrhythmia after myocardial infarction, which may be related to the activation of mechanosensitive channels. Ln et al. (2011) demonstrated that TREK-1 mRNA expression was significantly reduced in the left ventricular infarct region after MI, resulting in decreased potassium efflux, prolonged repolarization time, and prolonged APD. However, the heart increases TREK-1 mRNA expression and current density in the infarct border region through a compensatory mechanism. These results suggest that the changes of TREK-1 expression in different regions after infarction may be related to the occurrence of ventricular arrhythmias. In addition, the expression of TREK-1 in endocardium and epicardium itself is different (Joy et al., 2004; Wang et al., 2013). Therefore, the differential expression of TREK-1 in different regions after infarction makes different regions differently sensitive to mechanical stretch, which may trigger different electrophysiological activities of ventricular myocytes and cause ventricular arrhythmias, such as ventricular fibrillation.

In the permanent coronary artery ligation model, TREK-1 knockout mice showed longer QT interval, longer action potential duration, increased calcium consumption, larger infarct size, and more severe systolic dysfunction compared with wild type mice, which confirmed the protective role of TREK-1 in MIRI (Kamatham et al., 2019). However, other studies have shown that TREK-1 can inhibit the proliferation of cardiomyocytes (Yang et al., 2014). In the neonatal rat model of acute myocardial infarction, TREK-1 expression is increased in the endocardium of ventricular myocytes, and silencing TREK-1 helps to promote cardiomyocyte proliferation and reduce cell damage caused by hypoxia. This may be due to the incomplete differentiation of cardiomyocytes in neonatal rats, which is characterized by a state of hypometabolism and a highercardiac tolerance to acute hypoxia (Ostadal et al., 2014). However, the specific reasons need to be further studied. After myocardial infarction, the heart compensates to cause ventricular hypertrophy (Shervin Prince et al., 2022). Wang et al. (2013) found that in a rat model of ventricular hypertrophy, TREK-1 protein expression levels in the endocardial region were increased accompanied by an enhanced current, which may be related to the cardioprotective role of TREK-1 in cardiac hypertrophy by regulating potassium ion flow. In addition, under pressure overload conditions, the specific deletion of TREK-1 in cardiomyocytes caused cardiac insufficiency, while the specific deletion of TREK-1 in cardiac fibroblasts showed a protective effect, which helped to prevent further deterioration of cardiac function (Abraham et al., 2018). It can be seen that TREK-1 plays different roles in the heart at different stages of development. In addition, TREK-1 plays a protective role in mature heart disease models.

TREK-1 may play different roles in different tissues and injury models. The increased level of forkhead box transcription factor D3 (Foxd3) is observed in the rat model of spinal cord ischemia-reperfusion, which directly activates the targeted inhibition of KCNK2 by miR-214 and exacerbates spinal cord ischemia-reperfusion injury (SCII). Inhibition of Foxd3/miR-214/Kcnk2 axis and increase of KCNK2 expression can alleviate SCII (Li et al., 2020a; Li et al., 2020b). During MIRI, miR-214 can activate PI3K/Akt pathway by targeting PTEN to alleviate MIRI (Zhuang et al., 2022; Zhu et al., 2023). TREK-1 has been shown to play a protective role (Kamatham et al., 2019) in the process of ventricular remodeling in adult rats with MIRI, but whether TREK-1 is involved in the regulation of MIRI through MiR-214-related pathway has not been reported in the literature, which provides ideas for future research.

TREK-2(KCNK10 or K2P10.1) is less expressed in heart tissue and more expressed in the central nervous system (Darkow et al., 2023; Schmidt et al., 2017b; Wenli et al., 2002; Rivas-Ramírez et al., 2020; Lesage et al., 2000; Kisselbach et al., 2014). Early studies detected the presence of TREK-2 mRNA in zebrafish hearts and chicken embryonic hearts (Gierten et al., 2012; Zhang et al., 2008); In rat, mouse and human hearts, although TREK-2 is expressed in both atria and ventricles, the expression is extremely low and predominantly in the atrium (Liu and Saint, 2004; Schmidt et al., 2017b). Pranlukast has recently been identified as a novel activator of TREK-2 (Wright et al., 2019). The small-molecule compound ML335 also acts as an agonist of TREK-2, stabilizing TREK-2 conformation by binding at the “modulator pocket” generated by the displacement of the M4 helix; The activating nanoantibody CA10776 was shown to be highly selectivex for TREK-2 activation (Rödström et al., 2024). GI-530159 was identified as a selective TREK-2 inhibitor (Ajc et al., 2018). Knockdown of TREK-2 significantly attenuated the neuroprotective effect induced by isoflurane preconditioning in a rat model of middle cerebral artery occlusion, suggesting that TREK-2 plays an important protective role in cerebral ischemia (Zhao et al., 2019). However, the role of TREK-2 in cardiac disease has not been reported in the literature.

TRAAK(KCNK4 and K2P4.1) is mainly expressed in neurons (Fink et al., 1998), low expression in the heart (Howarth et al., 2018), and even there are reports that the expression of TRAAK cannot be detected in the heart (Darkow et al., 2023). The helical cap of TRAAK plays a vital role in the perception of chemical and mechanical stimuli (Brohawn et al., 2012). Aprepitant selectively activates TRAAK (McCoull et al., 2022). Vernakalant showed mild inhibition of human TRAAK (Seyler et al., 2014). Studies have shown that mice with TRAAK gene knockout can survive healthy after cerebral infarction and show a small ischemic area without significant arrhythmias (Lloyd et al., 2011). There are also research reports that TRAAK knockout can play a protective role in mouse cerebral ischemia (Laigle et al., 2012). As for the study of TRAAK in the heart, there are no reports in the literature.

### 2.3 TASK and MIRI

Members of the TASK subfamily include TASK-1, TASK-3, and TASK-5, which are sensitive to extracellular acidic environments (Duprat et al., 1997; Sindhu et al., 2000; Ashmole et al., 2001). In addition to pH responsiveness, TASK-1 and TASK-3 may form channel homodimer or heterodimers (Rinné et al., 2015), showing a variety of physiological functions.

TASK-1 (KCNK3 or K2P3.1) is found in the conduction system of the heart. TASK-1 expression is detectable in human and rat sinoatrial and atrioventricular nodes (Sh et al., 2011; Ferdous et al., 2016; Chandler et al., 2009). Early expression is also detectable in the whole heart of mice and chickens, but as the heart develops, its expression is eventually restricted to the His bundle, bundle branches, and Purkinje fibers (Graham et al., 2006). A large number of studies across species, including mice, rats, pigs, guinea pigs, as well as humans, have detected higher expression of TASK-1 in the atrium than in the ventricle (Liu and Saint, 2004; Wiedmann et al., 2018; Schmidt et al., 2014b; Schmidt et al., 2018; Skarsfeldt et al., 2016; Felix et al., 2020). Paradoxically, a small number of studies have reported that TASK-1 expression is more significant in the ventricle (Donner et al., 2011). However, we do know that the expression of TASK-1 channels is the highest in the heart and is expressed throughout the heart, regardless of whether the expression is higher in the atrium or the ventricle (Wiedmann et al., 2018).

TASK-1 is extremely sensitive to changes in pH. Under conditions of low extracellular potassium or acidification, cardiac TASK-1 channel open, triggering an increase in outward current (Kim et al., 1999). TASK-1 channel can be activated by halothane (Kiyoshi et al., 2006; Pandit et al., 2020) and inhibited by α1A-adrenergic receptors, doxapram, A293 and ML365. Among them, ML365 and A293 are specific inhibitors of cardiac TASK-1 channels (Caroline et al., 2007; Skarsfeldt et al., 2016; Felix et al., 2020; Wiedmann et al., 2022; Le Ribeuz et al., 2021).

The widening of QRS complex and prolongation of QT interval were observed in ventricular myocytes of TASK-1 knockout mice, which may be due to the decrease of potassium efflux during action potential repolarization, which prolongs APD (Pandit et al., 2020). Due to the fact that TASK-1 in mice is mainly expressed in the conduction system (Graham et al., 2006), changes in QRS complex and QT interval may also be caused by the obstruction of excitation conduction in some areas of the mouse ventricular muscle after TASK-1 knockout, leading to asynchronous excitation conduction in the ventricular muscle. In addition, after isoflurane anesthesia, the heart rate of wild-type mice decreased, while that of TASK-1 knockout mice did not change. The mechanism may be that isoflurane activates TASK-1 channels in the sinoatrial node and cardiomyocytes, resulting in increased potassium efflux and cell hyperpolarization at rest, which prolongs the time to the next excitation and leads to decreased heart rate. The prolongation of PR interval and the shift of Wenckebach point to longer cycle length indicate that the conduction efficiency of the atrioventricular node is reduced (Ieda et al., 2009; Dorninger et al., 2023). The reason may be that the activation of TASK-1 channels in the atrioventricular node also promotes potassium efflux, leading to cell hyperpolarization at resting potential and delaying the onset of the next action potential, which leads to the delay of atrioventricular conduction. Another study reported that the expression of TASK-1 was upregulated in the sinoatrial node of diabetic rats, and the heart rate was decreased in the langendorff *ex vivo* perfusion model of diabetic rats, indicating that TASK-1 is involved in the generation and conduction of electrical signals in the sinoatrial node (Ferdous et al., 2016). Notably, Donner et al. observed that TASK-1 knockout mice had increased mean heart rate and decreased heart rate variability, which were mainly due to the expression of TASK-1 in the heart and lung neurons (Donner et al., 2011). Recently, in a study to identify specific markers of lung pericytes by single-cell transcriptomics, high expression of TASK-1 was found (Sh et al., 2022). This suggests that TASK-1 in lung pericytes may be involved in the regulation of cardiac electrical activity and further explore the role of TASK-1 in the regulation of arrhythmias and the underlying mechanisms. Therefore, TASK-1 plays an important role in the ventricular repolarization process and excitation conduction of the heart in the resting state.

Under pressure overload, TASK-1 knockout mice have better cardiac function and reduced cardiac hypertrophy than normal mice, which may be related to the enhanced fatty acid oxidation by AKT phosphorylation and peroxisome proliferator-activated receptor-γ coactivator-1α, suggesting that TASK-1 may be involved in the pathophysiphysiological process of cardiac hypertrophy and heart failure (Duan et al., 2020). Left ventricular dysfunction is also associated with reduced atrial TASK-1 expression in patients with sinus rhythm (Schmidt et al., 2017a). Studies have reported that low concentrations of ethanol can play a protective rolex in MIRI by reducing oxidative stress through activation of aldehyde dehydrogenase 2 (ALDH2) (Kang et al., 2014). In the rat model of diabetic cardiomyopathy, ALDH2 activation by low concentration of ethanol may regulate the current and protein expression of TASK-1 to reduce myocardial injury and fibrosis, thus playing a protective role in diabetic cardiomyopathy (Zhang et al., 2019).

The TASK-3 (KCNK9 or K2P9.1) channel is mainly expressed in the human cerebellum, while its expression in the heart has not yet been clearly detected (Skarsfeldt et al., 2016; Ad et al., 2001). Only a few articles have reported extremely low expression of TASK-3 mRNA in the heart (Y et al., 2000). However, studies have reported that relatively high expression of TASK-3 can be detected in the right atrial appendage of rats and humans, and that expression of TASK-1/TASK-3 heterodimer, which has been shown to reduce affinity for TASK-1 blockers, can be observed in the heart or in heterologous expression systems (Rinné et al., 2015). Notably, the terbinafine and biguanide compounds CHET3 have been identified as selective activators of TASK-3 (Ping et al., 2019; Wright et al., 2017). Under stress and overload conditions, there was no significant difference in the transcription of fibrotic genes between TASK-3 knockout and TASK-1 knockout mice. Compared with wild-type mice, although the hemodynamics of cardiac function in both groups are basically similar, TASK-3 knockout mice have a slower onset of cardiac hypertrophy and dysfunction, indicating that TASK-3 may play a role in maintaining cardiac morphology while its impact on the progression of myocardial disease is limited (Duan et al., 2020).

As for TASK-5 (KCNK15 or K2P15.1), previous literature reported that its expression in the heart was extremely low, and no functional expression was observed in a heterologous expression system (Blin et al., 2014; Schmidt et al., 2015; I et al., 2001). These findings suggest that TASK-5 may have a relatively limited role in the heart, but its potential role in the physiological and pathological processes of the heart needs further investigation to clarify its function and importance.

### 2.4 TALK and MIRI

As a part of the K2P, the TALK family consists of three members: TALK-1, TALK-2 and TASK-2. There was a certain degree of sequence similarity between these members: TALK-1 and TALK-2 shared 44% sequence identity and TASK-2 shared 39% sequence identity; Sequence identity between TALK-2 and TASK-2 reached 37% (Girard et al., 2001; Reyes et al., 1998). In addition, the heterodimer TALK-1/TALK-2/TASK-2 is sensitive to the intracellular and extracellular alkaline environment and can regulate the cell membrane potential in response to large changes in extracellular pH (Khoubza et al., 2022; Decher et al., 2001; Mi et al., 2010).

TALK-1(KCNK16 or K2P16.1) is only expressed in the pancreas (Khoubza et al., 2022).

TALK-2 (TASK-4, KCNK17 or K2P17.1) is widely distributed in the human body, but its expression in the heart is restricted to the atrium and atrio ventricular node (Decher et al., 2001; Staudacher et al., 2018a; Staudacher et al., 2018b). The activity of TALK-2 channels can be modulated by a variety of compounds. Lidocaine and bupivacaine can inhibit TALK-2 channel (Kindler et al., 2003), while propafenone and propranolol can activate it (Staudacher et al., 2018a). Notably, NO can also activate TALK-2 (Duprat et al., 2005). A large number of studies have shown that NO can reduce oxidative stress by inhibiting the production of ROS, and reduce inflammatory response by inhibiting Nuclear factor erythroid 2-related factor 2 signaling transduction, thus playing a protective role in MIRI (Zhu et al., 2021; Hao et al., 2022; Hausenloy et al., 2017; Jacket et al., 2022). However, whether TALK-2 is activated by NO and involved in MIRI has not been reported in the literature. At present, there are few studies on cardiac TALK-2. It has been reported that TALK-2 has been shown to play an important role in ventricular repolarization in human pluripotent stem cells-induced cardiomyocytes, and its expression level is higher in non-ischemic heart failure tissues than in ischemic heart failure tissues (Chai et al., 2017). Therefore, TALK-2 may play a role in arrhythmia.

TASK-2 (KCNK5 or K2P5.1) is classified as a member of the TALK subfamily because it is more sensitive to alkaline environments (Reyes et al., 1998). The pH-gating mechanism of the TALK channel involves the changes of corresponding amino acids and protons inside and outside the cell to regulate the opening and closing of the channel (Li et al., 2020a; Li et al., 2020b). In the tissue expression profile, TASK-2 was highly expressed in the kidney (Reyes et al., 1998), while its expression in the heart was relatively low and mainly expressed in the atrium (Howarth et al., 2018; Syeda et al., 2016). TASK-2 channels are highly sensitive to local anesthetics and can be activated rapidly, which is mainly mediated by its carboxyl domain (Kindler et al., 2003). In addition, ROS can also activate TASK-2 channels and participate in the regulation of renal cell volume reduction during apoptosis (L’Hoste et al., 2007). TASK-2 expression is dysregulated in AF (Mondéjar-Parreño, 2022). Among the causes of decreased heart rate in the diabetic rat model, the expression of KCNK5 was found to be downregulated, suggesting that KCNK5 may play a role in controlling heart rate (Howarth et al., 2018). Fahima Syeda et al. (Syeda et al., 2016) found that TASK-2 may be involved in the paired-like homeodomain-2 transcription factor on atrial corrected resting membrane potential (RSP), thus serving as a target for Antiarrhythmic drug therapy, including atrial selective therapy. More and more studies have confirmed that TASK-2 is involved in the occurrence of arrhythmia, and whether it is involved in the pathophysiological process of MIRI requires more in-depth research on its role in heart diseases in the future.

### 2.5 THIK and MIRI

The THIK subfamily consists of two members: THIK-1 and THIK-2. THIK-1 and THIK-2 have 430 and 405 amino acids, respectively, and share 58% identity in amino acid sequence (Schmidt et al., 2015).

THIK-1(KCNK13 or K2P13.1) was the first channel isolated from rat brain and heart tissues (Rajan et al., 2001), and has been found in a variety of human tissues, including brain, heart, skeletal muscle, lung, kidney, liver, stomach, testis, and spleen (Schmidt et al., 2015). RNA and protein expressions of THIK-1 have also been detected in porcine hearts (Staudacher et al., 2019). Two variants of KCNK13, zkcnk13a and zkcnk13b mRNA, were detected in the brain and heart of zebrafish. Functionally, however, zkcnk13a is inactive, while zkcnk13b shows outward rectification. Human KCNK13 was similar to zebrafish with 70% to zkcnk13a and 66% to zkcnk13b (Ingo et al., 2019). The THIK-1 is differentially expressed in a variety of cell lines, including *xenopus* oocytes and HEK293T cells (Rajan et al., 2001; Kang et al., 2014). In addition, cardiomyocytes derived from patient-specific induced pluripotent stem cell also expressed THIK-1 (Chai et al., 2017). And its expression pattern is similar to that of human cardiomyocytes, it has been applied to many pathophysiological studies (Ingo et al., 2019; Zhao et al., 2021; Leigang et al., 2022; Jw et al., 2020; Elisa et al., 2020). Electrophysiological studies in heterologous expression systems have revealed the characteristics of the THIK-1: it has a small single-channel conductance, which is the smallest current generation in the known K2P channel (Kang et al., 2014). Although the inward current generated by it is weak, it can generate a strong outward current. The activity of THIK-1 is activated by AA, Gi/o-coupled receptors and Gq-coupled receptors, while it is inhibited by halothane, bupivacaine, quinidine, barium ion, and mexiletine (Rajan et al., 2001; Staudacher et al., 2019; Ingo et al., 2019; Kang et al., 2014). Small molecule compounds, such as CVN293 and C101248, have been recently developed to specifically block the THIK-1 in mouse microglia and inhibit the release of inflammatory mediators (Bürli et al., 2024; Ossola et al., 2023). Reduced release of IL-1β in microglia in the mouse THIK-1 knockout model also indicated that THIK-1 was involved in the inflammatory response (Gardener et al., 2004). In HE293 cells, THIK-1 has been proved to be sensitive to O2, which can be reversibly inhibited by acute hypoxia (Campanucci et al., 2005). THIK-1 is involved in the process of inflammation and oxidative stress, and these two processes are also important mechanisms of MIRI. Whether THIK-1 participates in MIRI through these processes has not been reported in the literature.

THIK-2(KCNK12 or K2P12.1) is strongly expressed in human stomach, liver, kidney and other tissues, especially in brain. However, the expression of THIK-2 has not been detected in the heart (Rajan et al., 2001). Human and rat THIK-2 is silent in *Xenopus* oocytes, and its current is difficult to detect (Bichet et al., 2015). Although THIK-1 and THIK-2 are highly homologous and can form an active heteromeric channel, addition of THIK-1/THIK-2 heterodimer to THIK-1 reduced the current of the channel (Blin et al., 2014).

### 2.6 TRESK and MIRI

TRESK (KCNK18 or K2P18.1) is found in brain, cerebellum, brain stem and spinal cord, as well as testis, among other tissues, but its expression in heart tissue has not been detected (Schmidt et al., 2015; Czirják et al., 2004). Therefore, the role of TRESK in the physiological or pathological processes of the heart is limited or has not been identified.

## 3 Prospect

The understanding of K2P channel family has changed from that background channel to that of pathophysiological state. However, the molecular mechanisms involved in K2P in cardiovascular diseases, especially in MIRI, are still poorly understood. Each K2P subfamily has different effects in MIRI, and the same member also shows different biological effects at different stages of heart development. Therefore, future studies need to explore and elaborate its related roles and specific mechanisms in more detail, in order to find and precisely regulate the targets of K2P in MIRI. In view of the changes in the expression of K2P channels in other diseases (such as Brugada syndrome), it is necessary to further study the role and specific mechanism of K2P channels in other cardiac diseases, so as to provide theoretical basis for clinical treatment.
